# Functional Characterization of an *Arabidopsis* Profilin Protein as a Molecular Chaperone under Heat Shock Stress

**DOI:** 10.3390/molecules27185771

**Published:** 2022-09-06

**Authors:** Hyosuk Son, Young Jun Jung, Seong-Cheol Park, Il Ryong Kim, Joung Hun Park, Mi-Kyeong Jang, Jung Ro Lee

**Affiliations:** 1 Department of Polymer Science and Engineering, Sunchon National University, Suncheon 38286, Korea; 2 National Marine Biodiversity Institute of Korea, Seocheon 33662, Korea; 3 National Institute of Ecology (NIE), Seocheon 33657, Korea; 4 Division of Applied Life Science (BK21+) and PMBBRC, Gyeongsang National University, Jinju 52828, Korea

**Keywords:** AtPFN, profilin, heat shock, higher molecular weight, molecular chaperone

## Abstract

Profilins (PFNs) are actin monomer-binding proteins that function as antimicrobial agents in plant phloem sap. Although the roles of *Arabidopsis thaliana* profilin protein isoforms (AtPFNs) in regulating actin polymerization have already been described, their biochemical and molecular functions remain to be elucidated. Interestingly, a previous study indicated that AtPFN2 with high molecular weight (HMW) complexes showed lower antifungal activity than AtPFN1 with low molecular weight (LMW). These were bacterially expressed and purified to characterize the unknown functions of AtPFNs with different structures. In this study, we found that AtPFN1 and AtPFN2 proteins have LMW and HMW structures, respectively, but only AtPFN2 has a potential function as a molecular chaperone, which has never been reported elsewhere. AtPFN2 has better protein stability than AtPFN1 due to its higher molecular weight under heat shock conditions. The function of AtPFN2 as a holdase chaperone predominated in the HMW complexes, whereas the chaperone function of AtPFN1 was not observed in the LMW forms. These results suggest that AtPFN2 plays a critical role in plant tolerance by increasing hydrophobicity due to external heat stress.

## 1. Introduction

The phloem is a living tissue in vascular plants that carries nutrients to all parts of the plant [1,2]. It is a transport system that functions as a conduit for viruses, RNA, proteins, lipids, and other small molecules [3,4,5]. Plant viruses, for example, target the phloem to rapidly establish a generalized infection; in so doing, they must overcome the host defense responses. Phloem-associated proteins that are effective at preventing virus movement play various roles in plant defense [4,5]. Therefore, the proper function of the plant phloem is essential for growth and development, as well as serving as a defense mechanism for biotic and abiotic stresses [6]. There have been many studies on immune defense mechanisms against the invasion of pathogens and external stress [7,8,9]. When a pathogen penetrates the plant tissue, potent defense molecules in the phloem cause cell death through the plasma membrane destruction of the pathogen. The phloem produces a variety of compounds, such as phytoalexin, antifungal peptides, and small proteins that inhibit the growth of pathogens [9,10,11].

Profilins were originally identified as being involved in actin polymerization [12,13,14,15], but they are also known to play an important regulatory role in F-actin dynamics [16,17]. A previous study confirmed that profilin-1 and -2 extracted from *Arabidopsis* phloem sap (AtPFN1 and AtPFN2, respectively) exhibited antifungal functions through apoptosis by inducing membrane potential breakdown and the release of cytochrome C in fungal cells [18,19,20]. It was also confirmed that AtPFN1 and AtPFN2 could inhibit fungal growth by producing cell-active oxygen species and mitochondrial superoxides [20]. In addition, profilin ligands, including phosphatidylinositol polyphosphate and proline-rich domain-containing proteins, have been identified [21]. The different affinities of profilins for these ligands are due to sequence differences between isoforms [21].

A previous study suggested exciting results from size-exclusion chromatography (SEC) analysis to confirm the purity of AtPFN1 and AtPFN2 proteins [20]. The study suggested that AtPFN1 and AtPFN2 proteins represent various oligomeric structures [20]. These results are similar to those reported in a previous study. In 1996, Babich et al. confirmed the presence of profilin dimers and tetramers [21,22], while the spontaneous oligomerization of profilin was reported by Wopfner et al. in 2002 [23].

Park et al. analyzed the secondary structures of AtPFN1 and AtPFN2 proteins using CD spectroscopy [20]. Although the α-helical content of AtPFN2 should be higher than that of AtPFN1 as predicted from their amino acid sequences, AtPFN2 showed strong antifungal activity in low-pH buffers by exhibiting a lower α-helical and higher β-strand content than AtPFN1. The antifungal activities of AtPFN1 and AtPFN2 proteins were measured using a microtiter plate assay. Through the MIC value, it was found that AtPFN2 was 2–4 times higher than that of AtPFN1 [20]. As indicated by their CD spectra, the difference in MIC values between AtPFN1 and AtPFN2 was speculated to be due to the structural difference between the two proteins. These features are well known as general functions of molecular chaperones. Therefore, based on a previous study, we performed a structural analysis of AtPFN1 and AtPFN2 to confirm if they are potential chaperone molecules.

This study investigated the molecular chaperone functions and structural changes in AtPFN1 and AtPFN2 in vitro. We focused on the relationship between the structures of AtPFN1 and AtPFN2 proteins and their functions. Biochemical evidence suggests that AtPFN2 may be involved in protein stability in a structure-dependent manner.

## 2. Results and Discussion

### 2.1. Stability of Arabidopsis Profilin Proteins in Plant Defense during External Heat Shock

**Figure 1:**
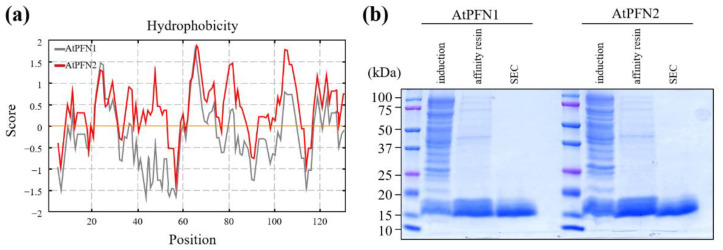
Comparison of the hydrophobicity of AtPFN proteins and their purification from *E. coli*. (**a**) The Kyte-Doolittle analysis generated hydrophobicity plots of AtPFN1 (**gray**) and AtPFN2 (**red**). The *y*-axis indicates the hydrophobicity scores; positive scores on the *y*-axis indicate hydrophobic regions. (**b**) Recombinant AtPFN1 and AtPFN2 were isolated via *E. coli* expression, and purity was confirmed using 13% SDS-PAGE and Coomassie blue staining. Induction, IPTG induced total proteins; affinity resin, a soluble protein purified by an affinity column; SEC, pure protein fractionated from SEC.

Hydrophobic binding is involved in conformational changes in proteins. Plant profilin proteins have been characterized through their binding affinities, subcellular distribution, and antifungal activity [20,24,25,26]. These results suggest that AtPFN proteins play essential roles in plant defense. However, the in vivo functions of *Arabidopsis* profilin proteins have not yet been extensively studied, except for their antifungal activities [20]. To characterize the structure and function of the two PFNs, their hydrophobicity was first confirmed based on their amino acid information (https://web.expasy.org/protscale/ accessed on 22 July 2022). It was confirmed that the hydrophobicity of AtPFN2 was higher than AtPFN1 (Figure 1a). In addition, we amplified the genes encoding these two proteins from *A. thaliana* and overexpressed two AtPFN proteins in *E. coli*. Finally, recombinant AtPFN1 or AtPFN2 proteins were purified using a His-tag affinity column and SEC analysis (Figure 1b).

**Figure 2:**
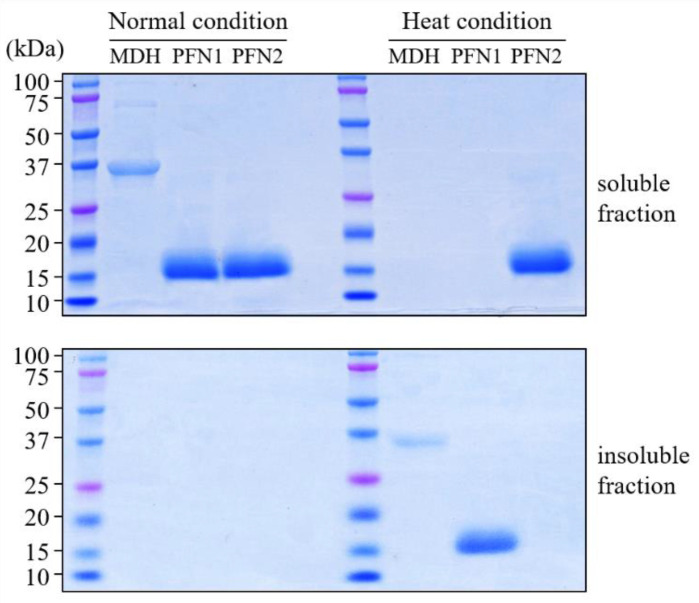
Stability of AtPFN proteins under heat shock conditions. Heat stability analysis of AtPFN proteins and malate dehydrogenase (MDH; control). Approximately 5 μg each of AtPFN and MDH were incubated at 25 °C (left; Normal condition) or 43 °C (right; Heat condition) for 30 min and then centrifuged at 13,000× *g* for 15 min. Each protein’s supernatant (soluble fraction) and pellet (insoluble fraction) were fractionated and analyzed using 13% SDS-PAGE.

According to previous reports, hazelnut profilin was reported to be heat-stable [27], while *Arabidopsis* PFN2 was reported to exist in a high molecular weight form [20]. To examine the heat-stability of the two *A. thaliana* PFN proteins, they were incubated for 30 min at 43 °C (Figure 2). Although the heat-sensitive proteins malate dehydrogenase (MDH) and AtPFN1 aggregated, AtPFN2 exhibited relatively improved heat stability, indicating solubility. Interestingly, AtPFN2 demonstrated better heat shock tolerance than AtPFN1 due to its higher molecular weight. A previous study elucidated the structural difference between profilin isoforms using SEC, CD spectroscopy, and *transmission electron microscopy* (TEM) [20]. These results suggest that AtPFN2 may have an additional role in plant stability under external heat stress.

### 2.2. Molecular Chaperone Function of AtPFN Proteins

**Figure 3:**
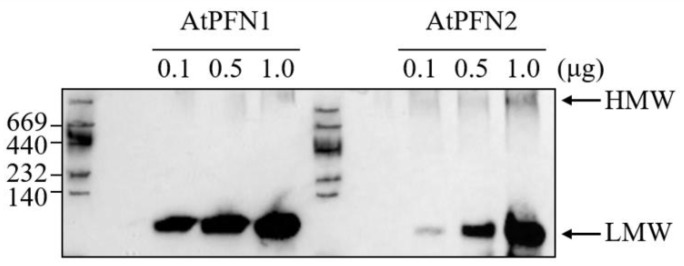
Structural analysis of recombinant AtPFN proteins in vitro. Recombinant AtPFN protein concentration-dependent aliquots were analyzed using a 10% native PAGE gel and silver staining. To confirm the native molecular mass of each AtPFN protein at normal condition, two recombinant proteins were analyzed via incubation at 25 °C for 30 min and centrifugation at 13,000× *g* for 15 min.

A previous study investigated the growth inhibition capacity of the two AtPFNs against several fungal cells and showed that AtPFN1 has a significantly higher antifungal activity than AtPFN2 [20]. Therefore, in the present research, the structural conformation of each PFN protein was firstly confirmed using 10% native PAGE (Figure 3).

**Figure 4:**
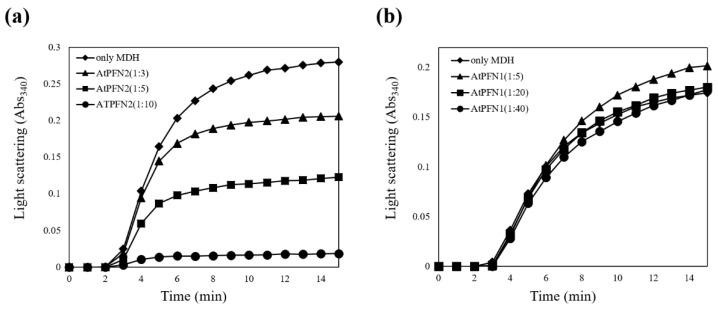
Comparison of holdase chaperone activity between AtPFN1 and AtPFN2. Thermal aggregation of 20 µg malate dehydrogenase (MDH) was examined at 43 °C for 20 min in the presence of AtPFN1 or AtPFN2 proteins. (**a**) Molar ratio of AtPFN2 to MDH: (▲) 3:1, (■) 5:1, and (●) 10:1. (◆) denotes the negative Control (MDH alone). (**b**) Molar ratios of AtPFN1 to MDH: (▲) 5:1, (■) 20:1, and (●) 40:1. (◆) denotes the negative control (MDH alone). Holdase chaperone activity by measuring the absorbance of the solutions at a wavelength of 340 nm.

The results confirmed that AtPFN2 had significantly more HWM forms than AtPFN1. HMW proteins are typically thought to protect denatured substrates from external stress, a characteristic of molecular chaperones [28]. The ability of the two AtPFN isoforms to prevent the heat aggregation of MDH was also explored to determine their molecular chaperone functions. Heat labile MDH was incubated with AtPFN1 and AtPFN2 at 43 °C, and heat-induced aggregation of the substrate was monitored using a spectrophotometer. Thermally denatured MDH has a higher absorbance as the degree of denaturation increases. When the chaperone protein inhibits the denaturation of MDH, the absorbance is lowered. The degree of decrease in MDH absorbance is indicative of chaperone activity. The unstable MDH was gradually protected upon incubation with AtPFN2 in a dose-dependent manner under heat shock conditions and was finally inhibited at a subunit molar ratio of 1 MDH to 10 AtPFN2 (Figure 4a). However, AtPFN1, due to its LMW structure, did not prevent the thermal aggregation of MDH (Figure 4b). These results suggest that AtPFN2 may have dual functions as an antifungal and molecular chaperone.

### 2.3. The Structural Difference between Profilin Isoforms

**Figure 5:**
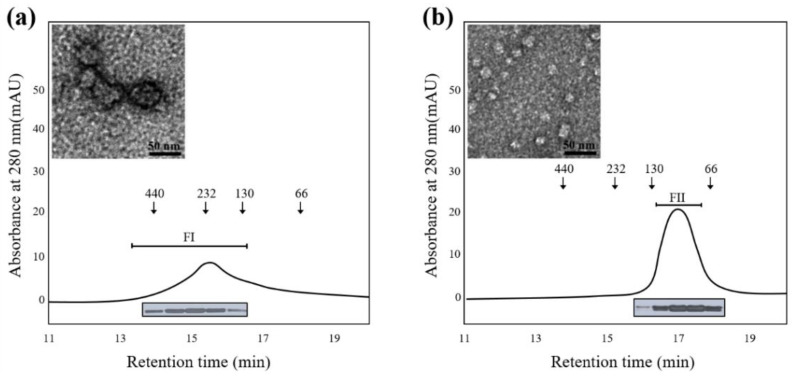
**Structural analysis of AtPFN2 via size-exclusion chromatography** (**SEC**) **and *transmission electron microscopy*** (**TEM**)**.** Purified recombinant AtPFN2 was separated using SEC based on MW. The HMW (**a**) and LMW (**b**) fractions of the AtPFN2 protein isolated through SEC were collected, and the structure of each fraction was determined. The HMW and LMW fractions were separated using 12% SDS-PAGE electrophoresis to confirm that they were indeed AtPFN2 proteins (inset). Oligomeric forms of AtPFN2 fractionated from SEC were observed under TEM (inset). The bar represents 50 nm.

**Figure 6:**
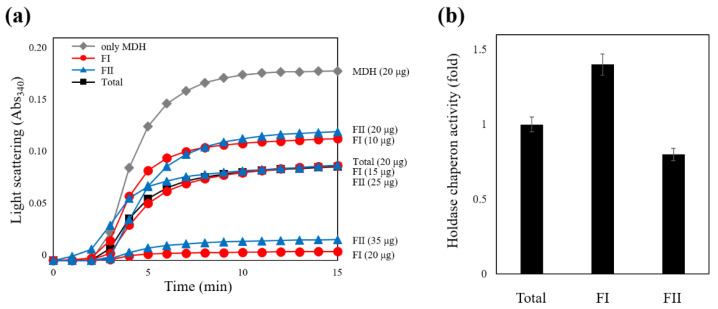
Association of AtPFN2 holdase chaperone function with protein structure. After equalizing the AtPFN2 levels in the two SEC fractions (FI and FII) and an aliquot of the total protein, the specific chaperone activity of AtPFN2 was measured using MDH as a substrate at 340 nm (A340). (**a**) Comparison of chaperone activity between FI and FII fractions of AtPFN2. Thermal aggregation of 20 µg MDH was examined at 43 °C for 20 min in the presence of FI or FII fraction of AtPFN2 protein. The activities of the different protein fractions were compared as a titration manner. (**b**) The activities of the different protein fractions were compared to those of the total protein. Total protein activity was measured under our assay conditions and set to 1 (fold). Representative results represent the mean of at least three independent experiments.

Many plant proteins with highly oligomeric structures function as molecular chaperones to protect cells from environmental stresses [28,29,30,31]. The structural enlargement of these proteins usually results from increased hydrophobicity due to various external factors [28,32,33,34]. It is well known that AtPFN proteins function as antifungal agents [20]. To determine the molecular size of the AtPFN2 protein with high chaperone activity, we expressed and purified soluble recombinant AtPFN2. First, total AtPFN2 protein was separated using SEC, the HMW and LMW fractions were collected, and SEC and TEM analyses were performed again. Interestingly, the AtPFN2 protein in the FI fraction obtained from SEC consisted of an oligomeric protein with a high molecular weight (HMW) with molecular masses ranging from about 130 to more than 440 kDa (Figure 5a). In contrast, proteins of the FII fraction had a lower molecular weight with masses of about 100 kDa (Figure 5b). Nevertheless, all protein fractions produced a single band with a MW of 14 kDa on an SDS-PAGE gel (Figure 5a,b, inset). These results suggest that AtPFN2 is a homo-oligomeric protein with various structures. Next, each protein fraction was collected and concentrated to investigate the structural dependence of the molecular chaperone function of AtPFN2. As shown in Figure 6, the FI fraction of AtPFN2 had more robust chaperone activity than the FII fraction. Specifically, the FI fraction exhibited approximately 1.4-fold higher chaperone activity than the unfractionated AtPFN2 protein, compared to the 0.8-fold chaperone activity of the FII fraction relative to the crude fraction.

**Figure 7:**
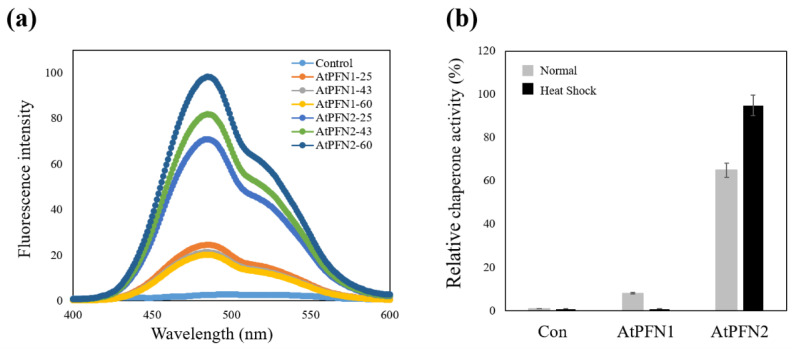
Comparison of the chaperone activity and hydrophobicity of AtPFN1 and AtPFN2 under heat shock conditions. Comparison of the chaperone activity and hydrophobicity of AtPFN proteins at different temperatures. (**a**) Hydrophobicity analysis under normal and heat shock conditions upon incubation with bis-ANS for 30 min at 25 °C, 43 °C, and 60 °C, respectively. The fluorescence of bis-ANS was measured using a fluorometer with an excitation wavelength of 390 nm and emission wavelengths of 430–630 nm. (**b**) Relative chaperone activities of AtPFN1 and AtPFN2 at 25 °C (normal) and 43 °C (Heat shock). Representative results represent the mean of at least three independent experiments.

**Figure 8:**
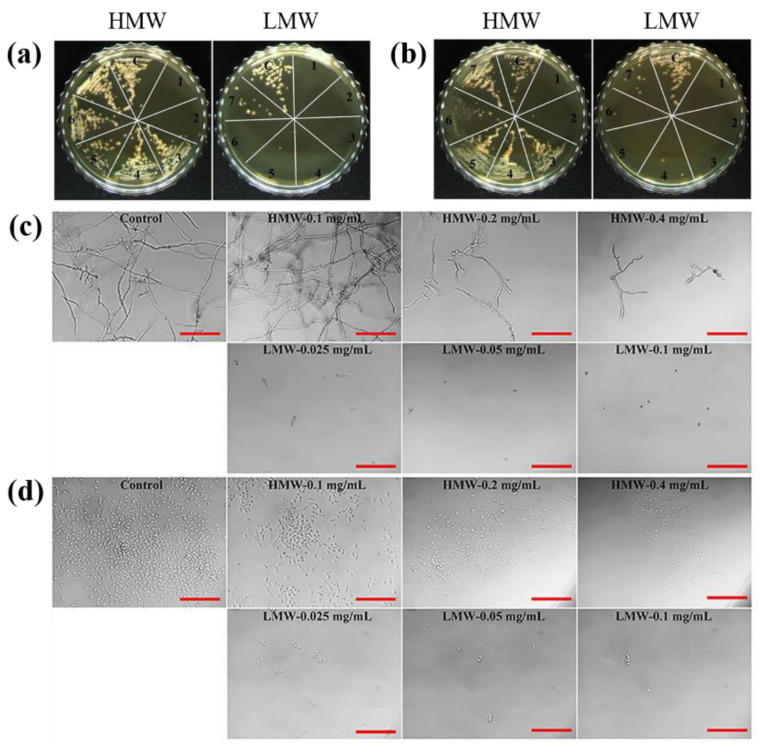
Antifungal activity of HMW and LMW fractions of AtPFN2 protein against four fungal strains. After 24 h incubation of fungal conidia ((**a**): *Candida krusei*, (**b**): *C. tropicalis*) and proteins, the solution was streaked on YPD agar, followed by additional 24 h incubation. c: control, 1: 0.5 mg/mL, 2: 0.25 mg/mL, 3: 0.125 mg/mL, 4: 0.0625 mg/mL, 5: 0.03125 mg/mL, 6: 0.0156 mg/mL, 7: 0.0078 mg/mL. After 24 h incubation of fungal conidia ((**c**): *Fusarium graminearum*, (**d**): *Cryptococcus* sp.) and proteins, the fungal growth was observed using a microscope.

Hydrophobic forces are essential for molecular chaperone activity and structural changes in proteins under external stress [30]. The hydrophobicity of AtPFN proteins was determined using bis-ANS probe, which binds to the hydrophobic regions. A spectrofluorometer was used to evaluate the bis-ANS binding, which demonstrated exposure of the hydrophobic region of AtPFN proteins. When the probe was bound to AtPFN proteins, its emission maximum was shifted. The increase in fluorescence intensity indicates more hydrophobic patches of AtPFNs exposed by heat treatment. The fluorescence intensity of the AtPFN2-bound bis-ANS probe was higher than that of AtPFN1, indicating that the hydrophobic regions of AtPFN2 were more exposed under normal conditions than those of AtPFN1 (Figure 7a). Furthermore, the hydrophobicity of heat-treated AtPFN2 was also compared to verify the correlation between the hydrophobic effect and structural changes in the protein (Figure 7a). It was confirmed that the degree of hydrophobicity of AtPFN2 increased than that under normal conditions. However, the hydrophobicity of AtPFN1 did not increase at various elevated temperatures (Figure 7a). It can be seen that the exposure of more hydrophobic residues in the AtPFN2 protein under heat shock increased the generation of the HMW complex via hydrophobic interactions [28,30]. In addition, it was confirmed that the holdase chaperone activity of AtPFN2 increased after heat treatment. Interestingly, fluorescence and chaperone activities continuously increased as the incubation temperature increased (Figure 7b). These findings confirmed our theory that AtPFN2 functions primarily as a molecular chaperone in HMW complexes (Figure 6a), whereas antifungal activity is present in LMW structures (Figure 8).

**Figure 9:**
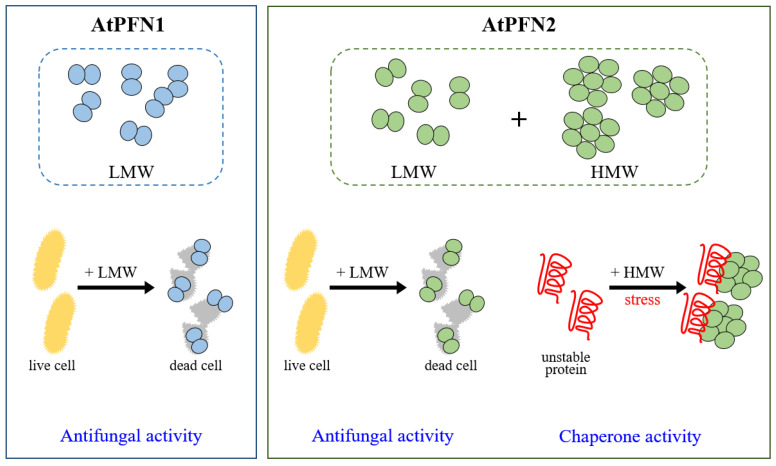
A representative model of oligomeric status and function of AtPFN proteins. AtPFN2 has dual functions as a chaperone and antifungal agent in the HMW and LMW structures, respectively. However, AtPFN1 with a LMW structure acts as an antifungal protein.

The present study revealed the unique physiological and molecular roles of AtPFN2, demonstrating that it functions as a molecular chaperone and antifungal protein to protect plants under various external conditions, such as heat shock and pathogenic attack (Figure 9). However, the mechanisms underlying the regulation of the structural and functional alterations of AtPFN2 have not yet been verified. Hence, more research is needed to determine how the protein structure of AtPFN2 is regulated compared to other *Arabidopsis* PFN proteins.

## 3. Materials and Methods

### 3.1. Materials

MDH and 1,1-bi-(4-anilinonaphthalene-5-sulfonic acid) (bis-ANS) were purchased from Sigma-Aldrich (St. Louis, MO, USA).

### 3.2. Purification and Structural Analysis of the AtPFN Proteins

Two AtPFN proteins were generated from an *Arabidopsis thaliana* cDNA library using PCR. The AtPFN genes were inserted into the overexpression vector (pET28 (a)) and transformed into *E. coli* BL21 (DE3). The transformed *E. coli* cells were grown on LB medium containing 50 μg/mL of kanamycin and induced with 1 mM isopropyl β-D-thiogalactopyranoside (IPTG) when the optical density at 600 nm (OD_600_) reached 0.6~0.8. Afterward, the cells were further grown at 30 °C for another 4~5 h. After harvesting the cells, the pellet was resuspended in lysis buffer (50 mM Tris-HCl, 200 mM NaCl, and protease inhibitor, pH 8.0). Meanwhile, the supernatant was centrifuged at 40,000× *g* for 30 min at 4 °C, filtered through a 0.45-μm filter, and then collected. AtPFN proteins bound to a HisPur™ cobalt resin (Thermo Fisher Scientific, Waltham, MA, USA) were eluted via imidazole treatment. Purified PFN proteins were then dialyzed against phosphate-buffered saline (PBS, pH 7.4) or 2-(N-morpholino)ethanesulfonic acid buffer (50 mM MES, 150 mM NaCl, pH 5.4). The purity of two PFN proteins isolated was determined using 12 or 13% SDS and 10% native-PAGE. The biochemical properties of the isolated proteins were analyzed as reported previously [20]. Two SEC fractions of AtPFN2 were put on carbon-coated grids (Harrick Plasma, Ithaca, NY, USA), and the grids were then negatively stained with 2% uranyl acetate. The structures of AtPFN2 were examined using a 200 kV FEI Tecnai 20 TEM, and their images were captured with a Gatan CCD camera.

### 3.3. Enzymatic Analyses of Molecular Chaperone and Antifungal Activity

Molecular chaperone activity was assayed using the model substrate MDH. Briefly, MDH was incubated in 50 mM HEPES-KOH (pH 8.0) buffer at 43 °C with various concentrations of AtPFN proteins. During a 20-min incubation, the thermal aggregation of MDH was determined by monitoring the increase in turbidity at A340 in a temperature-controlled spectrophotometer (DU800; Beckman), as described previously [29].

Growth inhibition assay of AtPFN2 against fungal pathogens was performed using previously reported methods [20]. *Candida krusei* (CCARM 14017), *C. tropicalis* (KCTC 7221), *Fusarium graminearum* (KCTC 16656), and *Cryptococcus sp.* (KCTC 17072) were obtained from from the Culture Collection of Antimicrobial Resistant Microbes (CCARM, Seoul Women’s University, Seoul, Korea) and Korea Collection for Type Cultures (KCTC, Jeongup-si, Jeollabuk-do, Korea).

### 3.4. Hydrophobicity Analysis of AtPFNs Using bis-ANS Fluorescence

The exposed hydrophobic regions of AtPFNs were examined by measuring bis-ANS binding to each FPLC fraction using an SFM 25 spectrofluorometer (Kontrom, Zurich, Switzerland), as described previously [29]. Reaction mixtures containing 10 μM of each fraction in 50 mM HEPES buffer (pH 8.0) were incubated with 10 μM bis-ANS at 25 °C, 43 °C and 60 °C for 30 min. The excitation wavelength of bis-ANS was set as 380 nm, and the emission spectra were scanned between 400 and 650 nm.

### 3.5. Size-Exclusion Chromatography and Polyacrylamide Gel Electrophoresis

Fast protein liquid chromatography (FPLC; Bio-Rad, USA) was performed using an Enrich size-exclusion chromatography (SEC) 650 column equilibrated with 50 mM 4-(2-hydroxyethyl)-1-piperazineethanesulfonic acid (pH 7.4) buffer at a flow rate of 0.5 mL/min at 25 °C. Protein fraction peaks (A280) were isolated and concentrated using a Centricon YM-10 unit (Millipore Co., Santa Clara, USA) [29,30]. Protein fractions obtained from the first SEC run were concentrated and stored at 4 °C until the second SEC was performed. SDS- and native-PAGE was performed using previously reported methods [28,30].

## Data Availability

Not applicable.
